# Macro-Microscopic Characterization and Long-Term Performance Prediction of Polyvinyl Chloride Under Hydrothermal Aging Based on Creep Behavior Analysis

**DOI:** 10.3390/polym17172320

**Published:** 2025-08-27

**Authors:** Hui Li, Xiaoxiao Su, Guan Gong, Aoxin Shao, Yanan Zheng

**Affiliations:** 1 College of Architecture and Civil Engineering, Xinyang Normal University, Xinyang 464000, China; su2301520392@163.com (X.S.); gongguan025@xynu.edu.cn (G.G.); s13653869318@163.com (A.S.); 2022241605@xynu.edu.cn (Y.Z.); 2 Henan New Environmentally-Friendly Civil Engineering Materials Engineering Research Center, Xinyang Normal University, Xinyang 464000, China

**Keywords:** hydrothermal aging, creep, accelerated characterization, local structural derivative Kelvin model, microstructure

## Abstract

The creep behavior of rigid polyvinyl chloride (PVC) in hydrothermal environments can compromise its long-term stability and load-bearing capacity, potentially leading to deformation or structural failure. Understanding this degradation is critical for ensuring the durability and safety of PVC in engineering applications such as pipelines and building materials. In this study, accelerated hydrothermal aging tests were carried out on PVC under controlled conditions of 60 °C and 90% relative humidity (RH). Short-term tensile creep tests at four different stress levels were conducted both before and after aging. Microstructural changes associated with the PVC’s creep behavior were analyzed using X-ray diffraction (XRD), Fourier transform infrared spectroscopy (FTIR), scanning electron microscopy (SEM), and other microscopic characterization techniques. These analyses provided a detailed microscopic interpretation of how hydrothermal exposure and applied loads influenced the macroscopic creep performance of the PVC, thereby elucidating the correlation between its macroscopic mechanical behavior and microstructural evolution. By applying the time–stress equivalence principle and the time–aging equivalence principle, the short-term creep behavior was characterized to predict long-term performance. The accelerated characterization curve can effectively predict the creep behavior of PVC under a stress level of 16 MPa over approximately 6.5 years in an environment of 60 °C and 90% RH. At the same time, the master creep modulus curve of PVC under any aging duration and stress level can be established under the specified environmental conditions of 60 °C and 90% RH. Long-term creep curves were fitted using a locally structured derivative Kelvin model, demonstrating that this model can effectively simulate the long-term creep behavior of PVC under hydrothermal conditions. The results indicate that at a stress level of 16 MPa, PVC is expected to undergo creep damage and failure after approximately 15 years in such an environment. These findings provide a critical reference for assessing the long-term performance of PVC in hydrothermal environments.

## 1. Introduction

Polyvinyl chloride (PVC) is one of the most widely produced plastic materials worldwide. Due to its excellent chemical stability, mechanical strength, and processing adaptability, it is extensively used in construction pipes (such as drainage and water supply pipes), packaging materials (including films and sheets), and electronic components [1]. PVC can be classified into rigid PVC, which exhibits high strength and rigidity and is primarily used in structural construction components, and flexible PVC, which offers good flexibility and is commonly employed in films and cable insulation layers. Understanding the changes in mechanical properties caused by aging is crucial to predict the service life of rigid PVC [2]. This study focuses on rigid PVC subjected to long-term exposure in hydrothermal coupling environments (such as temperature variations and moisture penetration in hot water pipes). Under this condition, the PVC material undergoes molecular chain hydrolysis and a dehydrochlorination reaction [3] due to hot aging, resulting in aggravated creep behavior and significant degradation of mechanical properties [4,5,6]. High humidity facilitates the infiltration of water molecules into the material, inducing molecular chain scission or crosslinking, which disrupts the original molecular structure. This time-dependent cumulative creep effect may result in material deformation that exceeds the critical threshold, ultimately leading to failure mechanisms such as viscoelastic deformation, rupture of primary or secondary bonds, shear yielding, crack propagation, and fracture [7,8,9,10]. Therefore, it is crucial to elucidate the mechanism underlying the evolution of its long-term mechanical properties.

It is important to highlight that experimentally determining the long-term creep behavior of materials presents significant challenges, as the service life of materials typically spans several years or even decades. Thus, accelerated characterization methods can be utilized to generate equivalent long-term creep data within a constrained timeframe [11,12,13,14]. In the accelerated testing approach, the degradation and failure of sample performance are evaluated under artificially controlled conditions. This method aims to efficiently assess product reliability by integrating modeling techniques, thereby significantly reducing the time required for experimental testing [15,16]. Among these methods, the Time–Stress Superposition Principle (TSSP) has been rigorously validated as an effective tool for predicting the long-term creep behavior of materials such as epoxy resin [17] and PTFE sealant [18]. Eftekhari et al. [19] combined the Time–Stress Superposition Principle (TSSP) with the Findley power law to model the nonlinear viscoelastic creep behavior and extrapolate long-term creep predictions from short-term experimental data for composite materials. Bennis et al. [20] conducted an accelerated hydrothermal aging experiment to validate the static damage law, demonstrating that the tensile strength of CPVC/PVC decreased by approximately 40% after hydrothermal aging at 80 °C.

Although accelerated characterization methods can effectively predict the long-term creep behavior of polymers based on the equivalence principle [21,22,23,24], the underlying evolution of macroscopic properties must still be elucidated through microstructural analysis, as the macroscopic creep behavior of polyvinyl chloride is fundamentally governed by changes in its internal microstructure. The accumulation of these microscopic changes leads to a progressive decline in macroscopic mechanical properties [25,26,27], which is manifested through characteristic deterioration features such as reduced strength and decreased elongation at break. Advances in analytical techniques, such as Fourier transform infrared spectroscopy (FTIR), scanning electron microscopy (SEM), and X-ray diffraction (XRD), have facilitated more accurate characterization of microstructural evolution in materials [28,29,30]. Saad et al. [31] investigated the thermal degradation behavior of unplasticized PVC by employing spectral analysis techniques. They implemented two distinct accelerated degradation conditions—low relative humidity (≤30% RH) and high relative humidity (60% RH)—to evaluate their effects on the material’s deterioration. Ouyang et al. [32] examined the microstructural alterations (e.g., changes in functional groups and crystallinity) of PVC microplastics subjected to UV aging, utilizing analytical methods such as SEM, FTIR, and XRD. These microstructural changes directly resulted in alterations in macroscopic properties, such as changes in surface morphology and mechanical behavior.

Polymer deformation can be mathematically described using combined models that integrate elastic and viscous elements [33]. This modeling approach offers an effective means of balancing experimental costs with the need for long-term predictions by quantifying the time-dependent behavior of materials. Several researchers have successfully achieved long-term extrapolation from short-term data through the integration of fractional derivative models with experimental techniques. For example, Yao et al. [34] integrated dynamic mechanical analysis (DMA) rheological experiments with the Maxwell model, the Kelvin–Voigt model, and the time–temperature superposition principle to effectively extrapolate short-term experimental data of PMMA type II for long-term predictions. Gao et al. [35] developed a Zener model based on fractional derivatives to quantify the frequency-dependent behavior of viscoelastic materials through relaxation and creep functions. They further extended the model’s capability to characterize material responses under complex stress states, thereby offering theoretical support for the refinement of related models. Fractional viscoelastic models have garnered considerable attention [36,37,38,39,40] due to their ability to capture complex power-law behaviors with fewer parameters. These models demonstrate superior parameter efficiency and descriptive accuracy in various applications, including copolymer-modified asphalt [41], engineering structures [42], and polymer stress relaxation [43].

**Figure 1:**
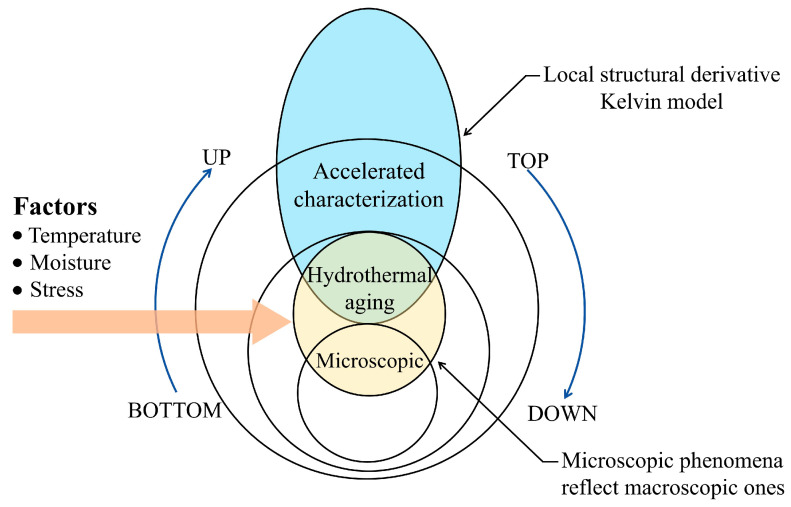
Research framework diagram.

However, the applicability of existing viscoelastic models remains limited under complex stress states and multiscale coupling conditions. Current research has primarily focused on the creep behavior of polymers under single-factor conditions, such as isolated stress or temperature fields, with a lack of systematic investigations into the combined effects of multiple factors in hydrothermal environments. Moreover, studies on the correlation between microstructural evolution and macroscopic creep behavior of PVC during thermo-moisture aging remain limited in depth and scope. To address these limitations, this study conducted accelerated aging tests on PVC under controlled hydrothermal conditions (60 °C, 90% RH) to evaluate short-term creep behavior. Microstructural changes before and after aging were analyzed using XRD, FTIR, and SEM. Long-term creep master curves were constructed based on the Time–Stress and Time–Aging Superposition Principles, and the long-term creep behavior was described through a localized structural derivative Kelvin model derived from the master curves. This study systematically investigated the creep behavior of materials under complex and extreme conditions involving multi-factor coupling of high temperature and high humidity. By integrating macroscopic mechanical creep testing with microscopic structural evolution analysis, this research aimed to uncover the intrinsic correlation mechanisms among environmental factors, creep deformation, and microstructural damage. The findings provide a more universally applicable and theoretically robust framework for predicting the long-term performance and assessing the service life of PVC materials in hydrothermal environments. The research framework is shown in Figure 1.

## 2. Materials and Experimental Methods

### 2.1. Material and Sample Preparation

**Figure 2:**
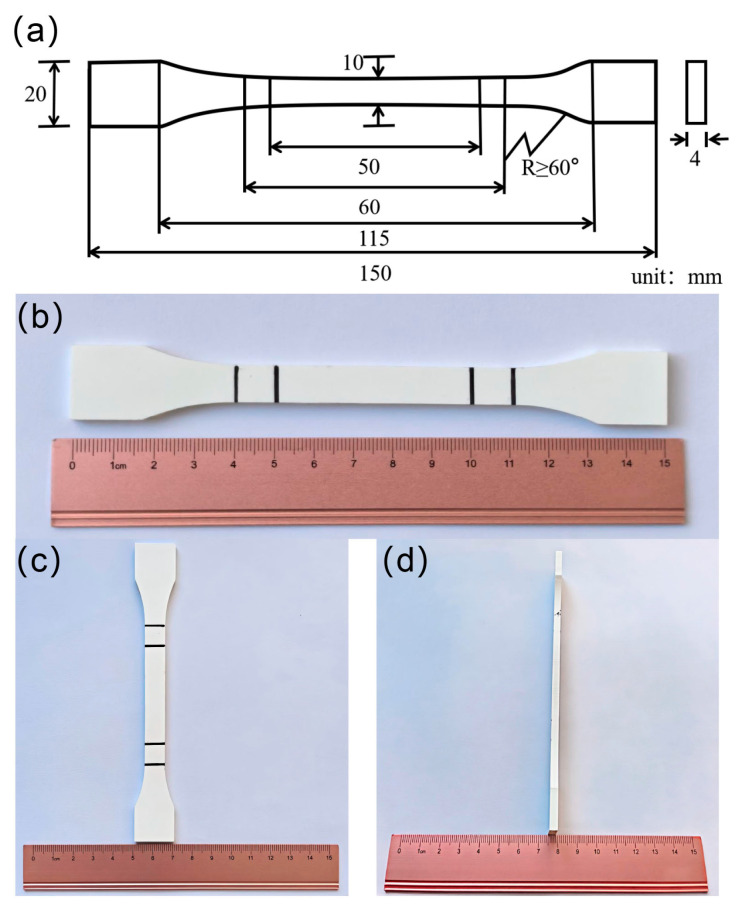
PVC specimen diagram: (**a**) dimensions of the specimen, (**b**) length of the specimen, (**c**) width of the specimen, (**d**) thickness of the specimen.

The material used in this study is rigid polyvinyl chloride (PVC), grade Trovidur^®^, supplied by Dongguan Xinweiteng Plastics Co., Ltd., Dongguan, China. The tensile tests of the specimens were conducted strictly in accordance with GB/T 1040.1-2018 [44] Determination of Tensile Properties of Plastics—Part 1: General Principles, with a testing speed of 5 mm/min. In compliance with GB/T 1040.2-2022 [45] Determination of Tensile Properties of Plastics—Part 2: Test Conditions for Moulded and Extruded Plastics, Type 1B dumbbell-shaped specimens were machined from extruded PVC sheets (thickness: 4.0 ± 0.2 mm). The key dimensional parameters are depicted in Figure 2a.

### 2.2. Aging Experiment in a Hydrothermal Environment

**Figure 3:**
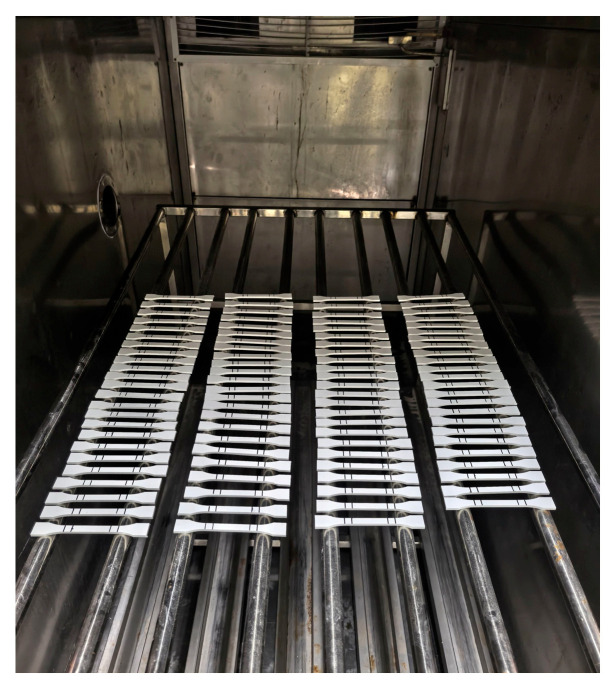
Hydrothermal aging test of PVC samples.

The equipment used in the hydrothermal aging experiment was an HR450P type high and low temperature alternating humidity and heat test chamber (Shanghai Hance Intelligent Technology Co., Ltd., Shanghai, China),which conforms to the IEC 60068-2-67 standard [46]. The test conditions were set at 60 °C with 90% RH. Five sample groups were established: one normal temperature control group (regulated according to Environment A in GB/T 2918-2018 [47]: temperature 23 ± 2 °C, relative humidity 50 ± 5%) and four experimental groups. As shown in Figure 3. Each group consisted of 20 specimens to ensure the statistical reliability of the results. The four experimental groups were evenly distributed within the hydrothermal environmental chamber. Thereafter, one group of specimens was removed from the chamber every 7 days for creep testing.

### 2.3. Tensile Creep Test

The creep test was conducted using a DWD-100E electronic universal material testing machine (Jinan Xinguang Testing Machine Manufacturing Co., Ltd., Jinan, China) equipped with a strain measurement device. The testing machine’s loading system employed closed-loop control technology to ensure the stability and precision of the applied stress. Tensile creep testing was conducted in compliance with ISO 899-1:2017 [48], which outlines standardized requirements for specimens, testing conditions, and data recording procedures under constant tensile stress (consistent with the Type 1B specimen dimensions employed in this study).

**Figure 4:**
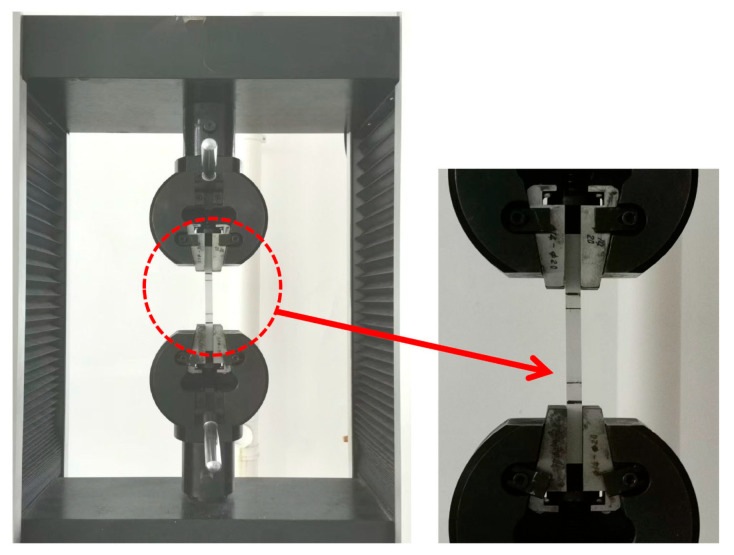
Tensile creep test of PVC specimens.

Figure 4 shows the tensile creep test of PVC specimens. In the context of PVC tensile creep testing, the selection of stress levels significantly influences test effectiveness. Excessively high stress levels may accelerate material failure, thereby compromising the reliability of the results; conversely, insufficient stress leads to negligible deformation, rendering the collected data insufficient and the test ineffective. To establish an appropriate stress range for the tensile creep tests, the tensile strength of PVC at room temperature was determined to be approximately 38 MPa, with an elongation at break of approximately 16%. Based on these baseline mechanical properties, a stress range corresponding to 36% to 52% of the material’s tensile strength was selected. Within this interval, four distinct stress levels—14 MPa, 16 MPa, 18 MPa, and 20 MPa—were chosen for testing. A 4 h tensile creep experiment was subsequently carried out to evaluate the time-dependent deformation behavior of PVC under sustained tensile loading within the defined stress range.

### 2.4. Microstructure Characterization

The crystalline structure of the PVC specimens before and after hydrothermal aging was analyzed using an X-Ray Powder Diffractometer (Smartlab9, Rigaku Corporation, Tokyo, Japan) with Cu Kα radiation (λ = 0.15418 nm). The device operated at 40 kV and 40 mA, scanning from 2θ = 10° to 90° at 10°/min. Molecular structural changes were evaluated using a ThermoFisher infrared spectrometer (Thermo Fisher Scientific Co., Ltd., Shanghai, China) integrated with an infrared microscope and a diamond ATR accessory. Spectra were collected between 4000 and 500 cm^−1^ at 4 cm^−1^ resolution, with each sample scanned 32 times to improve signal clarity. Samples were placed directly on the ATR crystal for analysis. Surface morphology was examined using a Hitachi S-4800 field-emission scanning electron microscope (FE-SEM, Hitachi High—Technologies Corporation, Tokyo, Japan), equipped with a cold field-emission electron gun, offering 1.0 nm secondary electron resolution at 15 kV and magnification from ×20 to ×800,000. Before imaging, samples were coated with a thin layer (~10 nm) of gold via ion sputtering to enhance conductivity.

### 2.5. Theoretical Analysis Methods

#### 2.5.1. Time–Stress Equivalence Principle

The Time–Stress Superposition Principle (TSSP) constitutes a pivotal theoretical framework for examining the long-term mechanical behavior of viscoelastic materials, such as polymers and composites, under complex stress conditions. This theory is fundamentally grounded in the free volume theory, which suggests [49] that the stress-induced change in the free volume fraction exhibits a linear relationship with the applied stress variation. The alteration in free volume directly impacts material mobility, thereby influencing its time-dependent mechanical properties [50]. Stress modifies the creep rate by altering the fraction (f) of free volume within the material [51], following the specific relationship below:

(1) $$f=f_{0}+\alpha_{\sigma }(\sigma -\sigma_{0})$$

where $f_{0}$ represents the free volume fraction under the reference stress, σ is the current stress applied, that is, the actual stress of the material, $\sigma_{0}$ is the reference stress, and $\alpha_{\sigma }$ denotes the stress sensitivity coefficient. An increase in the free volume within the material leads to a weakening of molecular interactions, thereby causing a reduction in material viscosity and an acceleration in the creep rate. The time–stress equivalence is given by the WLF equation through the horizontal shift factor ($log\alpha_{\sigma }$) [52]:

(2) $$\log\alpha_{\sigma }=-\frac{C_{1}(\sigma -\sigma_{0})}{C_{2}+(\sigma -\sigma_{0})}$$

where $C_{1}$ and $C_{2}$ represent material constants, which are determined through experimental calibration. If the material exhibits linear viscoelasticity (i.e., the stress–time equivalence demonstrates a linear relationship), then the simplified model must also be experimentally calibrated:

(3) $$\log\alpha_{\sigma }=\beta (\sigma -\sigma_{0})$$

where β denotes the linear stress sensitivity coefficient, which is defined by the relationship $\beta =(-C_{1}/C_{2})$.

Creep compliance, as an important indicator for measuring the deformation capability of materials under stress, can be expressed by the creep compliance J(σ,t) as follows:

(4) $$J(\sigma ,t)=J(\sigma_{0},\frac{t}{\alpha_{\sigma }})$$

The internal mechanism of stress acceleration characterization originates from the fact that stress modifies the characteristic time coordinate within materials. This phenomenon closely mirrors the creep process in viscoelastic materials, which is governed by the time–temperature equivalence principle. According to the time–stress equivalence principle, master curves at various stress levels can be constructed by horizontally shifting the creep experiment curves. The creep behavior and lifespan of materials can thus be predicted using a creep master curve with an extended time axis.

#### 2.5.2. Time–Aging Time Equivalence Principle

In addition to the time–stress equivalence principle, the time–aging equivalence principle plays a crucial role in studying material performance changes. By treating aging time as a factor that alters the relaxation spectrum of polymers [53], the Time–Aging Superposition Principle (TASP) was formally established. The time–temperature superposition principle describes the equivalent relationship between aging time and temperature effects on material properties during the aging process. During this process, both prolonged aging time and elevated aging temperature lead to variations in the free volume fraction within the material, thereby influencing its viscoelastic behavior. Based on the free volume theory, it is hypothesized that there exists a linear relationship between aging time and the free volume fraction of the material [54].

(5) $$f=f_{0}+\alpha_{H}(h-h_{0})$$

where f denotes the free volume fraction, h represents the aging time, $h_{0}$ signifies the reference aging time, and $\alpha_{H}$ corresponds to the expansion coefficient of the free volume fraction with respect to the aging time. The aging shift factor can thus be expressed as follows:

(6) $$\log\Phi_{H}=-\frac{C_{1}(h-h_{0})}{C_{2}+(h-h_{0})}$$

where $\Phi_{H}$ represents the aging shift factor, h denotes the actual aging time, $h_{0}$ corresponds to the reference aging time, and $C_{1}$ and $C_{2}$ are material-related constants associated with the rate of change of the free volume fraction.

The shift factor acts as the quantitative embodiment of the time–stress equivalence principle in practical applications, clarifying the relationship between material deformation behaviors under different stress levels.

#### 2.5.3. Local Structural Derivative Kelvin Model

The creep behavior is intricately associated with the accumulation of irreversible strain, where the viscoelastic strain can be represented as a nonlinear function of variables such as stress and time [55,56,57]. The creep behavior of polymers typically encompasses both reversible viscoelastic deformation and irreversible viscoelastic deformation. The viscoelastic component can be mathematically described using constitutive equations in either integral or differential forms, such as the following:

(7) $$\varepsilon (t)=\sigma_{0}+[J_{0}+\int_{0}^{t}J\left(t-\tau \right)\frac{d\sigma (\tau )}{d\tau }d\tau ]$$

Among them, ε(t) represents the strain of the material at time t, describing the deformation degree of the material at different moments. $J_{0}$ is the instantaneous compliance. J(t−τ) is the delayed compliance, which is a function of the time difference (t−τ). σ(τ) is a stress function with respect to time τ. t is the time variable for the current study. τ is an integration variable and serves as a dummy variable for time.

The traditional integer-order constitutive model has limitations in capturing the nonlinear creep behavior of materials under complex conditions. Incorporating fractional-order derivatives improves accuracy, especially for long-term deformation. The local structural derivative Kelvin model, an extension of the classical Kelvin model, uses local fractional-order derivative theory to better represent material characteristics during creep [58]. To analyze PVC creep in hot and humid environments, this model was used to fit the experimental data, providing a robust framework. Given a function $u\left(t\right)$ with time as the variable, its local structural derivative [58] is defined as follows:

(8) $$\frac{Su(t)}{St}=\underset{t^{\prime }\to t}{\lim}\frac{u(t)-u(t^{\prime })}{B(t)-B(t^{\prime })}$$

where B(t) represents the structure function. Specifically, when $B(t)=t^{\beta }$, the local structure derivative reduces to the fractal derivative; when B(t)=t, the local structure derivative reduces to the integer-order derivative.

**Figure 5:**
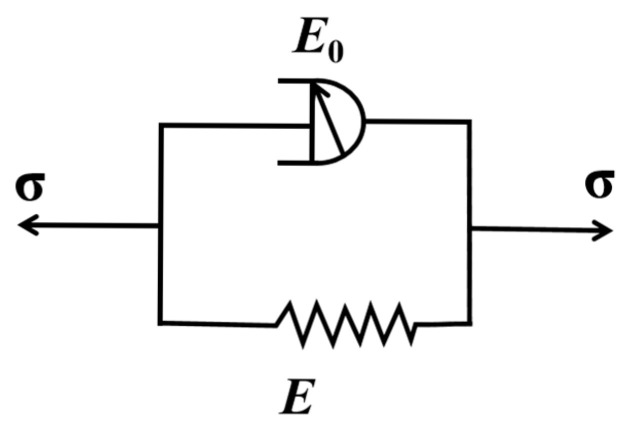
Schematic diagram of the Kelvin model for local structural derivatives.

In this paper, Equation (8) is utilized as the structural function for constructing the local structural derivative constitutive model:

(9) $$B(t)=\ln^{\alpha }(1+t/\tau_{0})$$

where α represents the order of the fractal derivative in time and $\tau_{0}$ represents the dimensionless representation of t. Within the framework of the local structural derivative modeling approach, the traditional dashpot is substituted with a structural dashpot, thereby yielding the corresponding local structural derivative viscoelastic model [58]. The constitutive relationship of the structural dashpot is defined as follows:

(10) $$\sigma =E_{0}\frac{d\varepsilon }{d\ln^{\alpha }(1+t/\tau_{0})}$$

where $E_{0}$ denotes the viscous modulus and α represents the order of the structural derivative. The local structural derivative Kelvin model can be derived by connecting the structural dashpot in parallel with the spring, as illustrated in Figure 5.

E is the elastic modulus. When the structural dashpot is connected in parallel with the spring, the total strain (ε) is equivalent to the sum of the strains in the viscous ($\varepsilon_{v}$) and elastic elements ($\varepsilon_{e}$), while the total stress is the summation of the stresses from both components, as follows:

(11) $$\varepsilon =\varepsilon_{e}=\varepsilon_{v}$$

(12) $$\sigma =\sigma_{e}+\sigma_{v}$$

It can therefore be concluded [59] that the constitutive equation described by the local structural derivative Kelvin model is as follows:

(13) $$E_{0}\frac{d\varepsilon }{d\ln^{\alpha }(1+t/\tau_{0})}+E\varepsilon =\sigma$$

According to the constitutive equation of the local structural derivative Kelvin model, when provided with the initial stress $\sigma_{0}$, it follows that

(14) $$\frac{d\varepsilon }{d\ln^{\alpha }(1+t/\tau_{0})}+\frac{E}{E_{0}}\varepsilon =\frac{\sigma_{0}}{E_{0}}$$

Let $\frac{E}{E_{0}}=C_{1},\frac{\sigma_{0}}{E_{0}}=C_{2}$, and obtain

(15) $$\frac{d\varepsilon }{d\ln^{\alpha }(1+t/\tau_{0})}+C_{1}\varepsilon =C_{2}$$

Separate the variables to obtain

(16) $$\frac{d\varepsilon }{C_{2}-C_{1}\varepsilon }=d\ln^{\alpha }(1+t/\tau_{0})$$

when t=0, ε(t)=0. From this, $C_{1}$ and $C_{2}$ are obtained. Then, integrating both sides simultaneously gives the creep process:

(17) $$\varepsilon (t)=\frac{\sigma_{0}}{E}(1-\exp(-\frac{E}{E_{0}}\ln^{\alpha }(1+t/\tau_{0})))$$

Creep compliance is

(18) $$J=\frac{\varepsilon (t)}{\sigma_{0}}=\frac{1}{E}(1-\exp(-\frac{E}{E_{0}}\ln^{\alpha }(1+t/\tau_{0})))$$

This represents the creep compliance expression of the local structural derivative Kelvin model.

## 3. Creep Test Results and Microscopic Characterization Analysis

Before delving into the creep behavior and microstructural changes of PVC, it is essential to clarify the evolution of its basic mechanical properties under hygrothermal aging conditions (60 °C, 90% RH). Table 1 presents the tensile strength and elongation at break of the PVC samples after different aging periods (0, 7, 14, 21, and 28 days), along with the standard deviation (SD) and coefficient of variation (CV%). The results reveal a consistent trend: as the aging time increases, both tensile strength and elongation at break exhibit a progressive and significant decline.

### 3.1. Characteristic Analysis of Material Creep Curves

**Figure 6:**
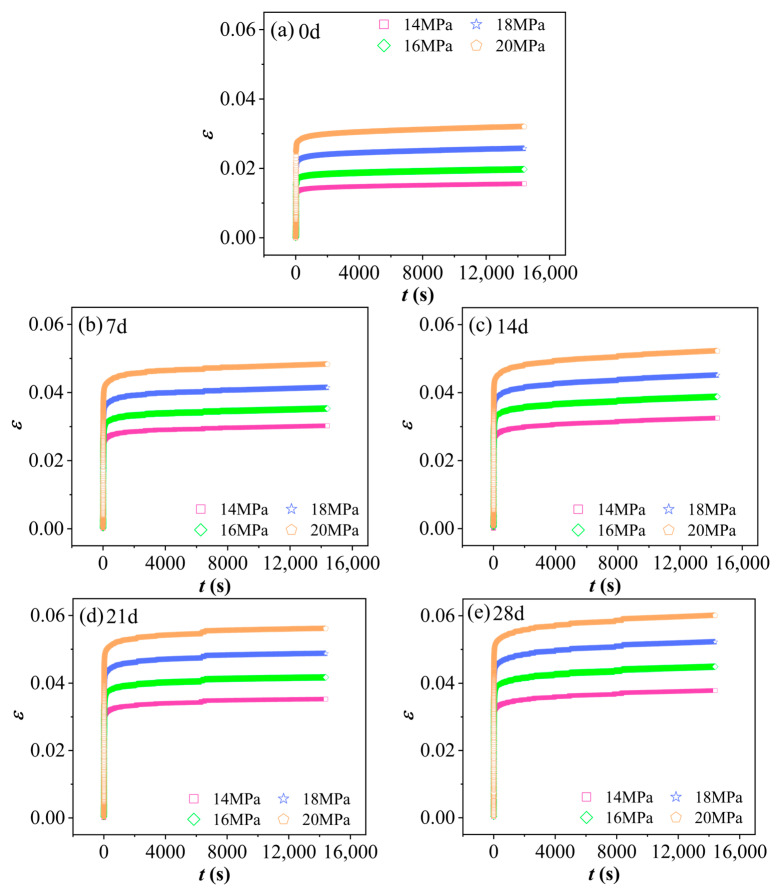
The ε−t curves of PVC under varying aging times and stress levels at 60 °C in a 90% RH environment. (**a**) 0 d (unaged), (**b**) 7 d, (**c**) 14 d, (**d**) 21 d, (**e**) 28 d.

Based on the definition that strain (ε) is the ratio of deformation to the original gauge length, the displacement data were converted into creep strain–time curves. Figure 6 presents the strain (ε)–time (t) curves of PVC under conditions of 60 °C and 90% RH at various aging times and stress levels. As shown in the figure, the curves represent only the primary creep stage and the steady-state creep stage; there is no indication of an accelerated creep stage. During the primary creep stage, strain increases rapidly over time, mainly due to the relaxation and reorientation of molecular chains within the material under applied stress. As time progresses, the material enters the steady-state creep stage, where strain exhibits an approximately linear relationship with time, resulting in a relatively constant creep rate. Refer to Table 2 for the steady-state creep rates of PVC at different aging times in a 60 °C and 90% RH environment. It can be observed that, for the same aging duration, higher stress levels lead to greater strain accumulation and faster creep progression. This is because higher stress promotes more rapid deformation and realignment of molecular chains, thereby accelerating the overall creep process.

**Figure 7:**
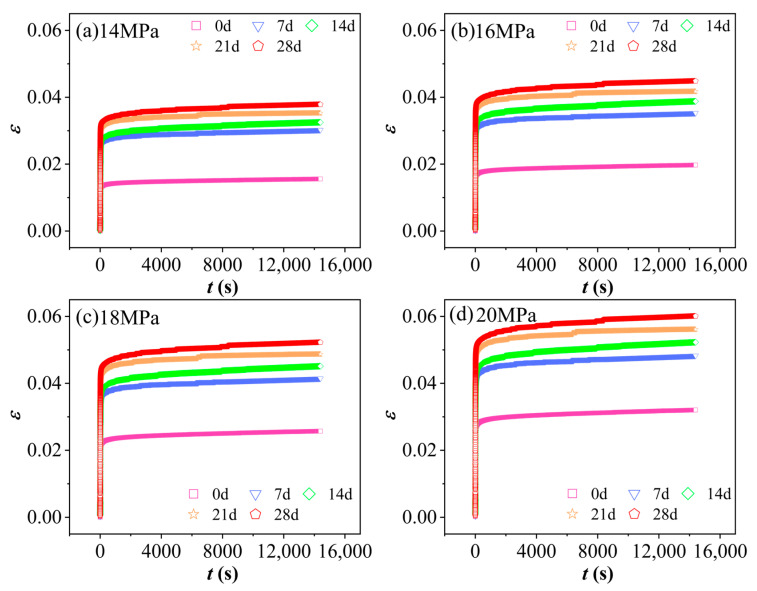
The ε−t curves of PVC at 60 °C and 90% RH under the same stress level but with varying aging times: (**a**) 14 MPa, (**b**) 16 MPa, (**c**) 18 MPa, (**d**) 20 MPa.

Figure 7 illustrates the ε−t curves of PVC at 60 °C and 90% RH under identical stress levels but varying aging durations. As shown in the figure, under a constant stress level, strain increases progressively with prolonged aging time. Furthermore, the creep deformation of specimens subjected to hydrothermal aging is significantly more pronounced than that observed at room temperature. This suggests that both humidity and temperature have a considerable influence on the creep behavior of PVC. Elevated temperatures enhance the material’s viscous flow characteristics, whereas increased humidity leads to a softening effect. The synergistic effect of these two environmental factors intensifies the viscoelastic response of the PVC. Consequently, the aged material exhibits more pronounced viscoelastic characteristics, as reflected by the continuous decline in the creep modulus and an increase in the steady-state creep rate. This trend results in a delayed onset of the accelerated creep stage.

**Figure 8:**
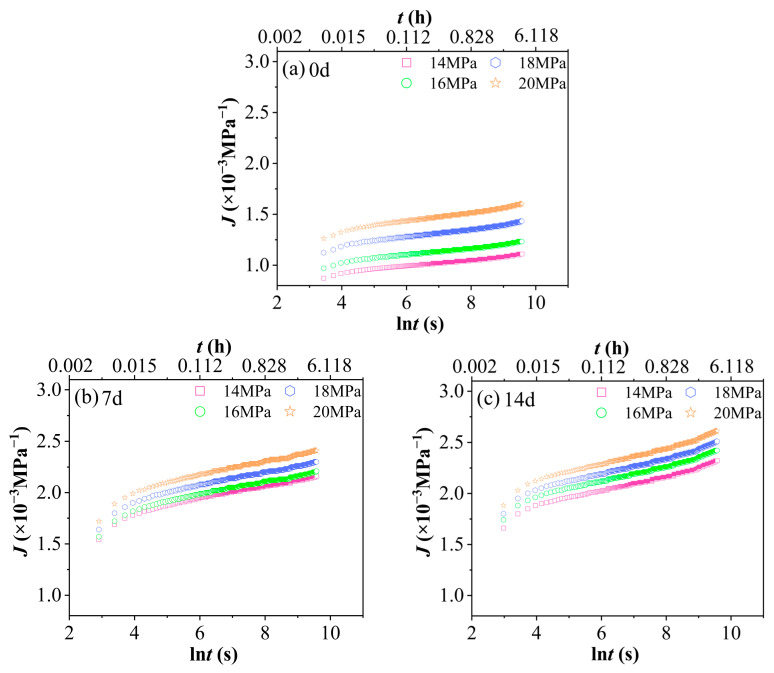

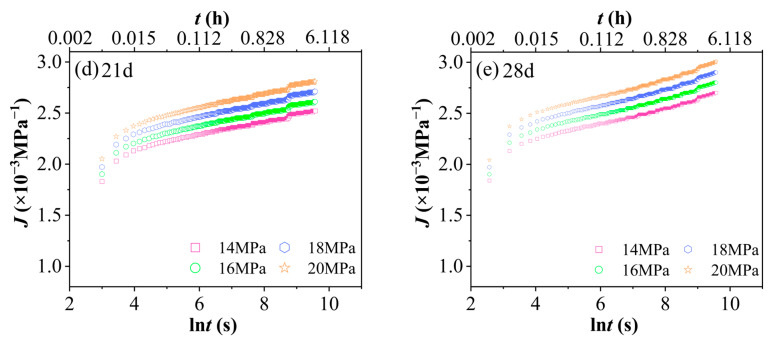
Creep compliance curves J−lnt of PVC at 60 °C and 90% RH under various aging durations and stress levels. (**a**) Unaged, (**b**) 7 d, (**c**) 14 d, (**d**) 21 d, (**e**) 28 d.

Creep compliance is defined as the ratio of strain to applied stress and serves as a quantitative indicator of a material’s deformation extent under unit stress. Figure 8 presents the creep compliance J–natural logarithm of time lnt curves for PVC after aging in a 90% RH environment under different stress levels. The horizontal axis represents the natural logarithm of time, lnt, in seconds, with values ranging from 2 to 10. The corresponding actual time, t, can be calculated as follows: when ln t = 2, t ≈ e2 ≈ 7.38 s (approximately 0.002 h); when lnt = 10, t ≈ e10 ≈ 22,026 s (approximately 6.12 h). This time range fully encompasses the duration of the 4 h short-term tensile creep test conducted in this study. Specifically, 4 h corresponds to ln t = ln (14,400) ≈ 9.57. Figure 8 clearly illustrates the inherent nonlinear viscoelastic behavior of PVC under hydrothermal aging, a key characteristic that is central to our accelerated characterization methodology. Importantly, the absence of superposition among the creep compliance ($J\left(t\right)$) curves at different stress levels (14, 16, 18, 20 MPa) across each aging state (0–28 days) confirms the material’s stress-dependent relaxation response. This lack of superposition not only aligns with expectations but also empirically supports the applicability and necessity of employing the Time–Stress Superposition Principle (TSSP) for predicting long-term performance in such complex environments. Moreover, the consistent increase in creep compliance with increasing stress for a given aging duration quantitatively indicates the progressive rise in molecular chain mobility and the activation of irreversible deformation mechanisms under higher loading conditions. Notably, this stress-dependent behavior, as shown in Figure 8, constitutes the critical experimental basis that enables the successful construction of stress-dependent master curves through horizontal shifting.

**Figure 9:**
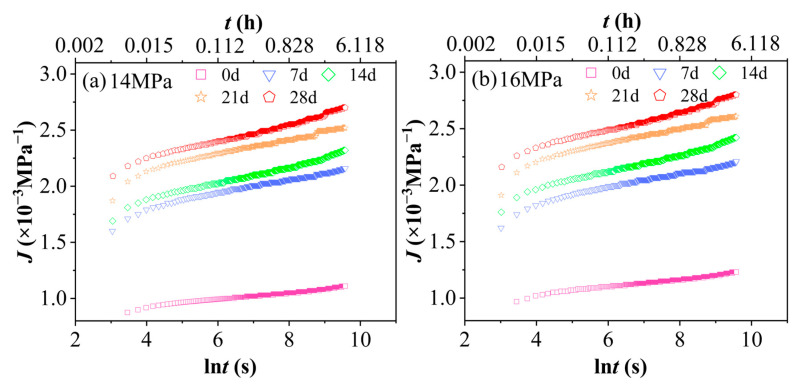

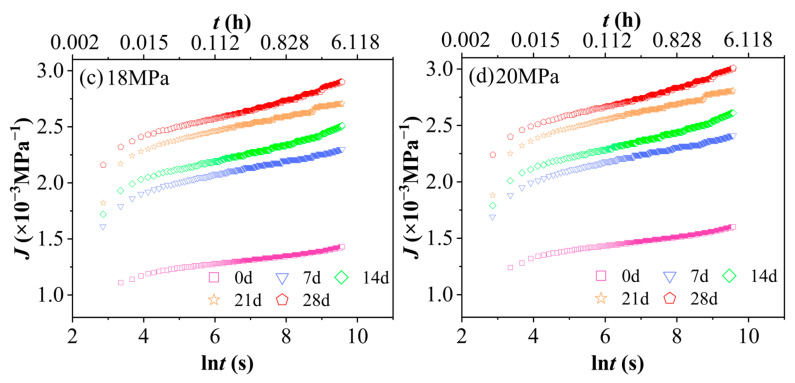
The creep compliance curves J−lnt of PVC at 60 °C and 90% RH under the same stress level but with varying aging times: (**a**) 14 MPa, (**b**) 16 MPa, (**c**) 18 MPa, (**d**) 20 MPa.

Figure 9 shows the curves J−lnt of PVC at 60 °C and 90% RH under a constant stress level with different aging durations. The horizontal axis, lnt(s), ranges from 2 to 10, corresponding to actual time values of approximately t ≈ 7.38 to 22,026 s (equivalent to about 0.002 to 6.12 h). This time range aligns with that of Figure 8. As illustrated in Figure 9, an increase in aging time results in a significant rise in the creep compliance (J) of the PVC. The hydrothermal-aged group demonstrates markedly higher creep compliance compared to the unaged group, indicating that PVC is considerably affected by hydrothermal conditions. This finding underscores the high sensitivity of PVC’s creep behavior to environmental changes during hydrothermal exposure.

### 3.2. Microscopic Morphology Analysis

#### 3.2.1. XRD Analysis

**Figure 10:**
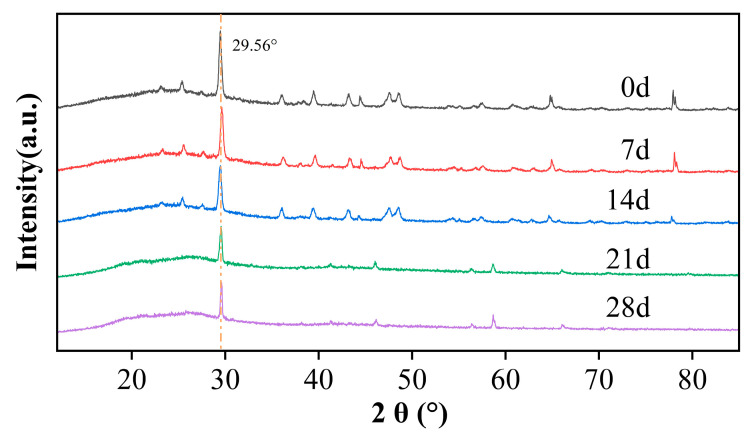
XRD patterns of PVC aged for different times in an environment of 60 °C and 90% RH.

The crystallinity of PVC is influenced by various factors, including molecular structure, molecular weight, polymerization method, and temperature, as well as its thermal and mechanical processing history (i.e., processing conditions). Thus, the crystallinity of PVC can vary over a wide range, which has triggered ongoing debates in academia regarding the exact values of PVC crystallinity and the interpretation of its XRD spectra [60,61,62]. Figure 10 illustrates the XRD patterns of PVC aged under conditions of 60 °C and 90% RH for varying durations. According to the XRD analysis, the crystalline structure of PVC undergoes slight degradation during hygrothermal aging at 60 °C and 90% RH. The characteristic peak of the (110) main crystal plane (2θ = 29.56°) appears as a sharp, high-intensity peak in the unaged state. With prolonged aging time, the amorphous region gradually expands, and background scattering in the low-angle region (20–25°) increases, indicating molecular chain relaxation and structural disordering. The intensity of the main diffraction peak decreases slightly from the unaged state to 28 days, suggesting a limited reduction in crystallinity. Additionally, the intensity of the weak diffraction peak near 17.8°, corresponding to the (010) crystal plane, diminishes, implying local disruption in the lateral stacking order of molecular chains. Macroscopically, this reduction in crystallinity results in a significant decrease in the modulus of the PVC, which aligns with findings from previous macroscopic creep tests.

#### 3.2.2. FTIR Analysis

**Figure 11:**
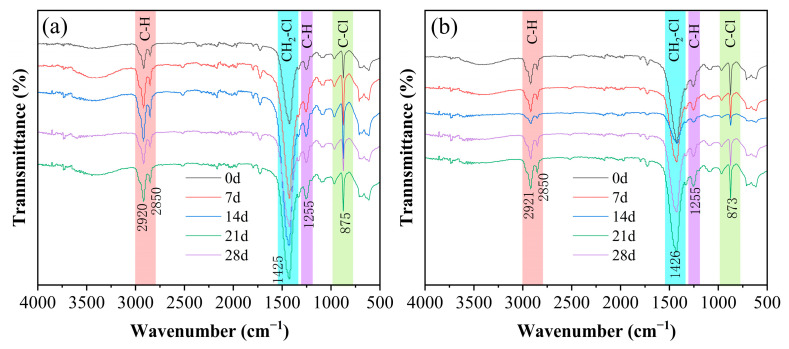
(**a**) FTIR spectra of PVC samples aged at 60 °C and 90% RH for various durations; (**b**) FTIR spectra of PVC aged at 60 °C and 90% RH, followed by 4 h creep deformation under 20 MPa stress.

Figure 11a illustrates that under conditions of 60 °C and 90% RH, as the aging time of the PVC sample increases, the positions and intensities of multiple characteristic peaks in the FTIR spectra undergo significant changes. The area of the C–H bond stretching vibration peak at 2800–3000 cm^−1^ gradually increases, indicating a reduction in absorption intensity and a decrease in the content of -CH_2_ groups within the aliphatic chain structure. In the 1400–1500 cm^−1^ region, the peak area initially increases and subsequently decreases, suggesting a decline in the content of -CH_2_-Cl groups. During the early stage of dehydrochlorination (de-HCl), the local regularity of the PVC molecular chain improves, resulting in an increase in peak intensity, which corresponds to the initial aging phase. However, as aging progresses, chain segment breakage disrupts the structural order, leading to a plateau or even a reduction in peak intensity. The turning point, occurring around 14 days, aligns with the time when the peak intensity at 1255 cm^−1^ reaches its maximum, implying that the aging mechanism transitions from being primarily dominated by de-HCl to a more complex process involving both chain scission and crosslinking. The peak intensity in the 1200–1300 cm^−1^ region remains relatively stable, although a slight shift in peak position is observed, indicating alterations in the chemical environment surrounding the bonds without a significant change in their reactivity. Meanwhile, the peak area in the 800–900 cm^−1^ region shows a slight decrease, which can be attributed to the dehydrochlorination reaction and is consistent with the reduction in crystallinity observed in the XRD analysis. Figure 11b illustrates that after a 4 h creep test under a stress of 20 MPa, the characteristic peaks of the PVC samples aged at 60 °C and 90% RH exhibit further changes as follows: in the 2800–3000 cm^−1^ range (C–H stretching vibration region), both peak intensity and area decrease, which can be attributed to molecular chain slippage induced by tensile creep, thereby altering the C–H vibration modes (a physical effect). The peak intensity in the 1400–1500 cm^−1^ range initially decreases and then increases, suggesting that the molecular chains become disordered at the onset of creep and later reorganize into a new ordered structure. Fluctuations in peak intensity and position within the 1200–1300 cm^−1^ range reflect the influence of varying intermolecular forces on chemical bonds. The peak in the 800–900 cm^−1^ range shows minimal change in intensity, indicating that external loading has a moderate impact on the molecular structure without causing significant damage. Notably, the attenuation slopes of the peaks at 875 cm^−1^ (C–Cl) and 2921 cm^−1^ (C–H) are significantly greater than those observed under pure hygrothermal aging conditions, suggesting mechanical chain scission in the PVC molecules. Taken together, these findings indicate that exposure to hygrothermal environments compromises the chemical structural stability of PVC. At the macroscopic level, this degradation manifests as a reduction in both tensile strength and elongation at break of PVC materials (Table 1). This decline in performance underscores the substantial influence of hygrothermal conditions on the mechanical properties of the PVC, rendering these materials more susceptible to deformation and damage under prolonged stress.

#### 3.2.3. SEM Analysis

**Figure 12:**
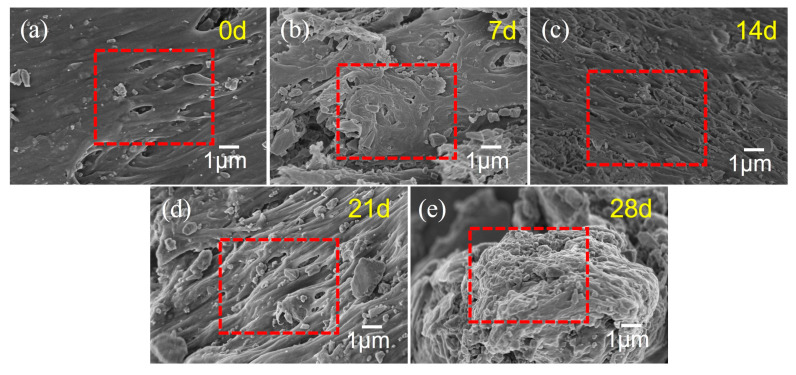
SEM images of PVC after aging under conditions of 60 °C and 90% RH for various durations: (**a**) 0 d, (**b**) 7 d, (**c**) 14 d, (**d**) 21 d, (**e**) 28 d.

Figure 12 displays the SEM images of the PVC after aging for various durations at 60 °C and 90% RH. At 0 days of aging, the PVC surface appears relatively smooth, with only a few minor protrusions and fine pores, which are characteristic features of the initial processing stage. As the aging period increases from 7 days to 28 days, the PVC surface progressively becomes rougher, exhibiting irregular gullies and large pores that indicate the formation of a loose, porous, sponge-like structure. The phase region structure formed by the aggregation of PVC molecules shows clear boundaries and contrasts between different phase regions, suggesting that moisture penetration in a hot and humid environment promotes reactions such as dehydrochlorination. Hydrogen chloride serves as a catalyst for molecular chain degradation, while the temperature of 60 °C accelerates the reaction rate, thereby enhancing the degradation and fracture of molecular chains on the PVC surface. Moreover, due to the motion and reorganization of the molecular chains, the PVC surface morphology undergoes gradual changes. Initially small particles either coalesce or migrate, and subtle undulations or wrinkles appear on the surface. These alterations can be attributed to the non-uniform distribution of surface stress caused by internal structural adjustments. Such microscopic structural changes lead to a decline in the macroscopic mechanical properties of the PVC, resulting in reduced strength and toughness. Additionally, the presence of numerous pores increases the external contact area, accelerating aging and reducing chemical resistance and water resistance.

**Figure 13:**
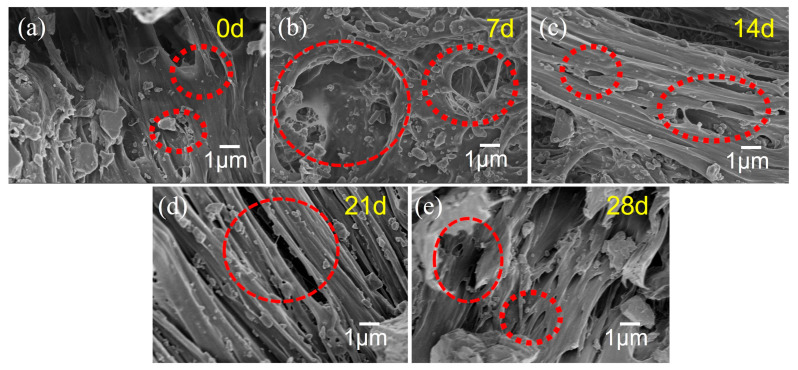
SEM images of PVC after aging at 60 °C and 90% RH for different times and creep test at 20 MPa stress for 4 h: (**a**) 0 d, (**b**) 7 d, (**c**) 14 d, (**d**) 21 d, (**e**) 28 d.

Figure 13 displays the SEM images of the PVC after aging for various durations under environmental conditions of 60 °C and 90% RH, followed by creep deformation under a stress of 20 MPa for 4 h. As shown in Figure 13a, the microstructure of the unaged PVC demonstrates a relatively regular pattern, with both the surface and internal structure being highly compact. The material exhibits minimal defects or cracks, indicating excellent structural integrity. After aging for 7 days, as illustrated in Figure 13b, the PVC material shows the formation of holes and structural changes. This suggests that under the specified conditions of 60 °C and 90% RH, the molecular chains of the PVC began to degrade, resulting in the relaxation of its physical structure. The SEM image after 14 days of aging (Figure 13c) reveals an increase in the number of holes, with fine cracks starting to appear in certain regions. Moreover, the microstructure has become more porous, and the degradation of the molecular chains has progressed further. Consequently, the internal defects within the material have proliferated. When aged for 21 days and 28 days, the cracks exhibit further propagation and widening. Additionally, the pores show a tendency to increase in number and connectivity, leading to a significant deterioration of the PVC microstructure. These observations align well with the aforementioned macroscopic creep test results.

## 4. Theoretical Modeling of Long-Term Creep Behavior and Life Prediction

### 4.1. The Equivalence Principles of Time–Stress and Time–Aging Time for Accelerated Characterization

**Figure 14:**
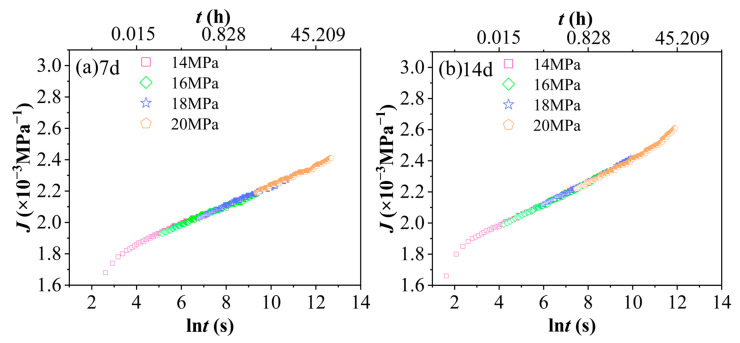

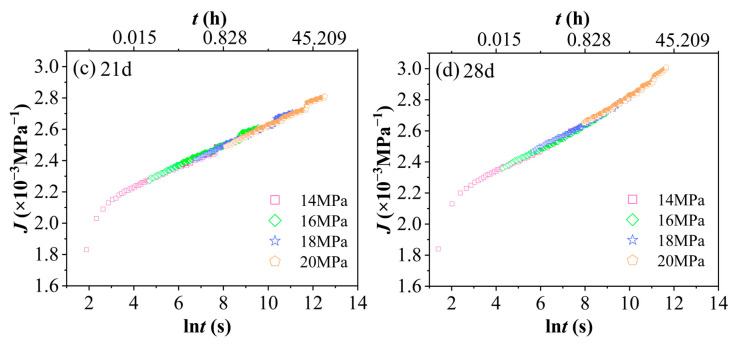
Master curves J−lnt of PVC aged at 60 °C and 90% RH for various durations, with a reference stress of 16 MPa: (**a**) 7 days; (**b**) 14 days; (**c**) 21 days; (**d**) 28 days.

**Figure 15:**
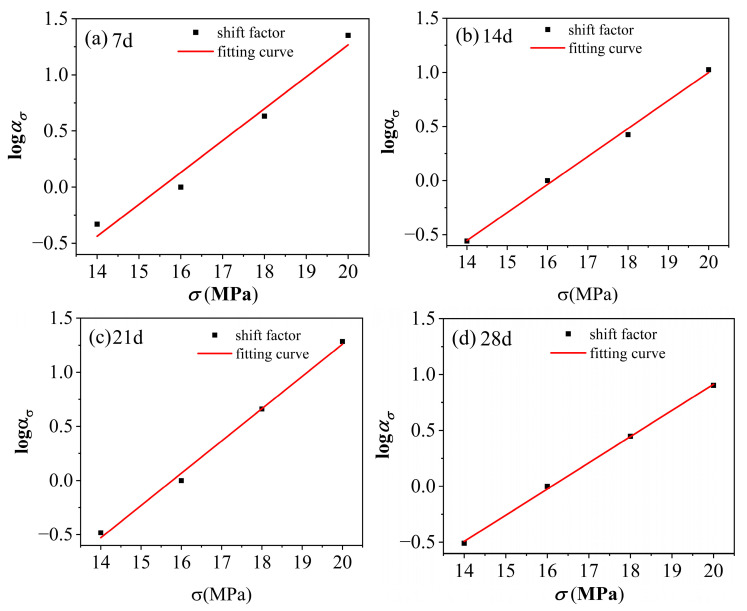
Fitting diagram of the shift factor for PVC aged under conditions of 60 °C and 90% RH at a reference stress of 16 MPa for various durations; (**a**) 7 days; (**b**) 14 days; (**c**) 21 days; (**d**) 28 days.

By leveraging the time–stress equivalence principle, the curves obtained under hydrothermal conditions shown in Figure 8 were accelerated and characterized. With a reference stress of 16 MPa selected, the curves corresponding to various stress states were horizontally shifted to align with the curve at the reference stress level. The specific shift factors for each curve are listed in Table 3—Horizontal shift factors at the reference stress of 16 MPa under different aging times at 60 °C and 90% RH. Consequently, the master curves J−lnt for PVC at 60 °C and 90% RH, accounting for different aging durations and utilizing a reference stress of 16 MPa, were successfully constructed, as depicted in Figure 14—Master curves J−lnt of PVC aged at 60 °C and 90% RH for various durations, with a reference stress of 16 MPa. When lnt ≈ 2 to 12.28, t ranges from 7.39 to 216,000 s. In other words, the time span corresponding to the range of lnt from 2 to 12.28 is approximately 60 h, which represents the long-term time predicted after acceleration. From the figure, it can be observed that the time span of the accelerated characteristic curve is approximately 15 times longer than that of the experimental curve, extending from an original duration of 4 h to 60 h. This adjustment significantly reduces the required experimental time. Moreover, as illustrated in Figure 15—Fitting diagram of the shift factor for PVC aged under conditions of 60 °C and 90% RH at a reference stress of 16 MPa for various durations, when the time–stress equivalence principle is applied for accelerated characterization, the shift factors corresponding to the translation of curves under the other three stress levels and various aging times to the reference curve approximately align along a straight line. The fitted linear equation is provided in Table 3. It can be seen that the shift factors for PVC under an environment of 60 °C and 90% RH, after aging for 7, 14, 21, and 28 days at any stress level, can be derived from the displacement factor equation. Using these shift factors, the creep compliance master curves for PVC under the same environmental conditions after aging for 7, 14, 21, and 28 days at any stress level can also be obtained.

**Figure 16:**
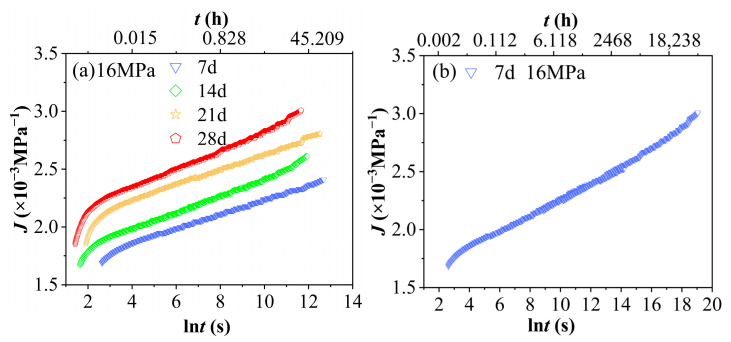
(**a**) The master curves J−lnt of PVC aged for various periods under conditions of 60 °C, 90% RH, and a stress of 16 MPa. (**b**) The master curve J−lnt of PVC aged for 7 days under conditions of 60 °C, 90% RH, and a stress of 16 MPa.

**Figure 17:**
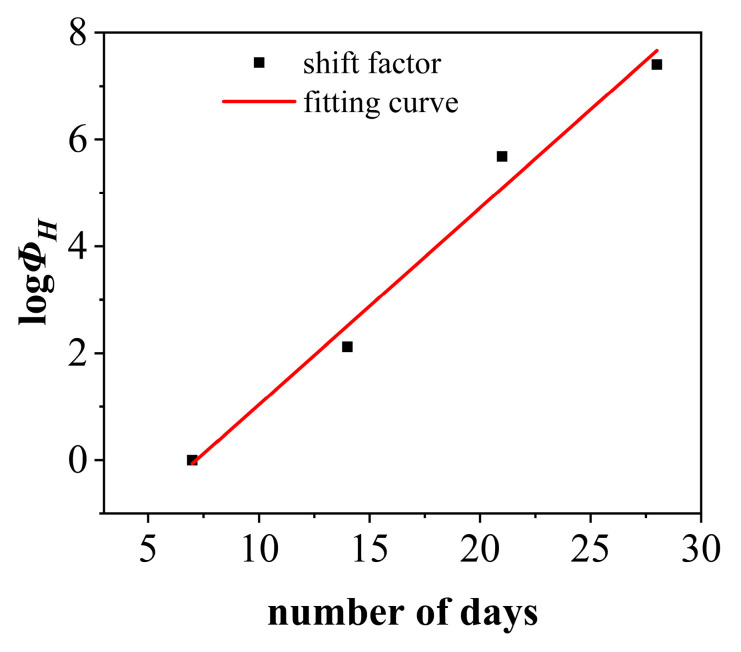
Fitting diagram illustrating the shift factor of PVC aged under a 60 °C, 90% RH environment with a reference stress of 16 MPa for varying aging periods.

Further, the time–aging equivalence principle can be utilized to conduct an accelerated characterization of the creep compliance master curve for PVC under conditions of 60 °C and 90% RH, with varying aging durations and a stress level of 16 MPa (as shown in Figure 16a). By selecting an aging time of 7 days as the reference point and aligning the other curves accordingly, it is possible to extrapolate a prolonged time–domain creep curve for PVC under the same environmental conditions after aging for 7 days at a stress level of 16 MPa, as depicted in Figure 16b. The range of the accelerated curve corresponds to lnt ≈ 2.6 − 19.14, which translates to a time range of approximately 13.46 s to 205,303,514 s, covering a time span of about 6.5 years. The corresponding time domain of this curve is expanded by a factor of 14,400 compared to the creep compliance experimental curve obtained after aging for 7 days at 60 °C and 90% RH under a stress of 16 MPa. This implies that the 4 h creep curve can be used to predict the creep behavior of PVC approximately 6.5 years after aging under the same conditions of 60 °C, 90% RH, and a stress level of 16 MPa. Meanwhile, upon recharacterization of the PVC using the time–aging equivalence principle under accelerated conditions, its displacement factor (as detailed in Table 4) still exhibits an approximately linear distribution, as illustrated in Figure 17. Therefore, the shift factors of PVC at any aging time under environmental conditions of 60 °C and 90% RH, as well as at any stress level, can be determined. Furthermore, the creep compliance master curve of PVC under the same environmental conditions and stress levels can also be constructed, significantly reducing the number of experimental conditions and the required testing time.

The linear fit of the time–aging shift factors ($\log\Phi_{H}$ versus aging time) presented in Figure 17 demonstrates a high coefficient of determination (R^2^ = 0.992). This strong linear relationship across the examined aging periods (7, 14, 21, and 28 days) suggests that the Time–Aging Superposition Principle can be consistently and reliably applied to predict long-term creep behavior based on short-term test results under the given hydrothermal conditions (60 °C, 90%RH, 16 MPa). Moreover, the close agreement of the shift factors corresponding to different aging times along the same linear trend further confirms the validity and consistency of the accelerated characterization approach across the studied aging states.

### 4.2. Model Simulation

**Figure 18:**
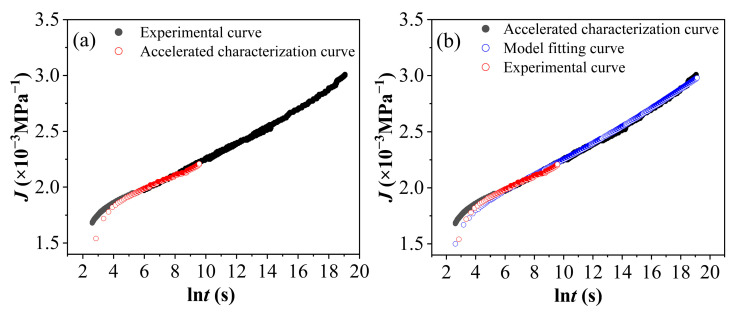
Comparison chart of curves for PVC aged under a 60 °C, 90% RH environment, with a reference stress of 16 MPa for 7 days. (**a**) Comparison between the test curve and the accelerated characterization curve; (**b**) comparison among the test curve, the accelerated characterization curve, and the model simulation curve.

Figure 18a presents a comparative chart illustrating the test curve and the accelerated characterization curve of PVC under environmental conditions of 60 °C and 90% RH for 7 days, with a reference stress of 16 MPa. As depicted in the figure, the accelerated characterization curve is in good agreement with the experimental curve. Figure 18b provides a comparative chart showing the test curve, the accelerated characterization curve, and the model simulation curve of PVC aged for 7 days under conditions of 60 °C and 90% RH, with a reference stress of 16 MPa. Notably, the primary curve in Figure 16b was fitted using the local structural derivative Kelvin model. The fitting results are presented in Figure 18b. Comparative analysis among the test curve, the accelerated characterization curve, and the model simulation curve yields a high coefficient of determination (R^2^) of 0.998924 for the fitted curve, indicating an excellent goodness-of-fit. Based on these fitting results, the model curve demonstrates excellent consistency with the experimental data curve, and the root mean square error is relatively low. This suggests that the model can accurately describe the creep behavior of PVC in hot and humid environments. Using the local structural derivative Kelvin model, it can be calculated that when PVC undergoes aging for 7 days in an environment of 60 °C and 90% RH, followed by creep under a stress of 16 MPa, the strain value will reach approximately 0.12456 after about 15 years, leading to creep fracture of the material.

## 5. Conclusions

In summary, this paper systematically investigated the long-term creep behavior of PVC in hot and humid environments. Through a series of experiments and detailed analyses, the following conclusions were drawn:

1. A hydrothermal aging test was conducted under conditions of 60 °C and 90% relative humidity, followed by short-term tensile creep tests at various stress levels. The results indicate that, for a given aging duration, higher stress levels lead to greater strain accumulation. Similarly, under the same stress level, prolonged aging time also results in increased strain accumulation. The combined effects of temperature and humidity synergistically intensify the viscoelastic response, leading to a reduction in the creep modulus and an increase in the steady-state creep rate. As stress increases, along with extended aging time and the influence of hydrothermal conditions, creep compliance rises significantly, highlighting the material’s high sensitivity to environmental changes.
2. Microscopic characterization was conducted using advanced microscopic techniques, including SEM, XRD, and FTIR. The results demonstrated a clear correlation between the evolution of the microstructure—specifically the expansion of amorphous regions, increased surface porosity, and alterations in functional groups—and the observed decline in macroscopic creep modulus as well as an increase in the steady-state creep rate. Such correlations represent a hallmark of polymer aging under hot and humid conditions. This study establishes a microscopic mechanistic foundation for interpreting the changes in macroscopic creep behavior.
3. This study validated the effectiveness of the time–stress equivalence principle and the time–aging equivalence principle under a hygrothermal coupling environment. By selecting appropriate reference stress and reference aging time, the short-term experimental data obtained within 4 h were effectively extrapolated to predict the long-term (approximately 6.5 years) creep behavior. This approach provides a reusable methodological framework for predicting the long-term performance of polyolefin viscoelastic materials. More importantly, when these equivalence principles were applied under accelerated aging conditions, the resulting shift factors exhibited an approximately linear distribution. This suggests that, based on the methodology verified in this study, the shift factors for polyvinyl chloride materials can be determined across different aging times, environmental conditions, or stress levels. Consequently, the creep compliance master curve can be constructed under the selected reference conditions (i.e., reference environment and stress level), significantly reducing both the number of required experimental conditions and the time needed to obtain long-term creep data.
4. The local structural derivative Kelvin model was employed to fit the long-term creep behavior of PVC, effectively capturing the time-dependent deformation characteristics under high-temperature and high-humidity conditions. The high goodness-of-fit (R^2^ ≥ 0.99) of the model for PVC creep data under the coupled effects of heat and humidity confirmed its suitability for this system and indicated its potential for simulating the creep behavior of polymers under multi-factor environmental coupling (e.g., heat, humidity, and stress). However, its applicability across different material systems requires further investigation and validation.
5. In future research, we plan to expand the range of experimental temperatures and humidities to obtain more comprehensive data and verify the universality of the conclusions. Furthermore, multiple models will be integrated for comparison with the local structural derivative Kelvin model, enabling a detailed evaluation of the advantages and limitations of each model in characterizing the creep behavior of PVC. This strategy is intended to furnish robust theoretical and methodological support for research in this field and facilitate the broader and safer application of PVC materials in engineering contexts.

## Figures and Tables

**Table 1:** Tensile strength and elongation at break of PVC at different aging times under 60 °C and 90% RH.

Aging Time (Days)	Tensile Strength (MPa)	SD	CV (%)	Elongation at Break (%)	SD	CV (%)
0	38.02	±0.8	2.1	16.0	±0.5	3.1
7	36.22	±0.7	1.9	14.4	±0.6	4.2
14	35.12	±0.9	2.6	12.8	±0.4	3.1
21	34.04	±1.1	3.2	11.6	±0.6	5.2
28	32.35	±1.3	4.0	10.6	±0.8	7.5

Table notes: Standard Deviation (SD)—measures the degree of dispersion of data relative to the average; Coefficient of Variation (CV)—the ratio of the standard deviation to the mean, which compares the degree of dispersion between different data sets.

**Table 2:** PVC steady-state creep rate at different aging times in 60 °C and 90% RH environment.

Aging Time (Days)	Stress (MPa)	Mean Creep Rate (h^−1^)	SD	CV (%)
0	14	3.96 × 10^−4^	3.37 × 10^−5^	8.5
	16	5.02 × 10^−4^	4.67 × 10^−5^	9.3
	18	6.54 × 10^−4^	6.67 × 10^−5^	10.2
	20	8.09 × 10^−4^	9.78 × 10^−5^	12.1
7	14	6.55 × 10^−4^	6.23 × 10^−5^	9.5
	16	7.64 × 10^−4^	6.71 × 10^−5^	8.8
	18	8.97 × 10^−4^	9.43 × 10^−5^	10.5
	20	1.04 × 10^−3^	1.25 × 10^−4^	12.0
14	14	9.55 × 10^−4^	7.83 × 10^−5^	8.2
	16	1.13 × 10^−3^	1.30 × 10^−4^	11.5
	18	1.31 × 10^−3^	1.18 × 10^−4^	9.0
	20	1.52 × 10^−3^	1.52 × 10^−4^	10.0
21	14	7.75 × 10^−4^	6.74 × 10^−5^	8.7
	16	9.13 × 10^−4^	8.95 × 10^−5^	9.8
	18	1.07 × 10^−3^	1.20 × 10^−4^	11.2
	20	1.23 × 10^−3^	1.27 × 10^−4^	10.4
28	14	1.00 × 10^−3^	9.19 × 10^−5^	9.2
	16	1.19 × 10^−3^	1.00 × 10^−4^	8.4
	18	1.38 × 10^−3^	1.49 × 10^−4^	10.8
	20	1.59 × 10^−3^	1.83 × 10^−4^	11.5

Table notes: Standard Deviation (SD)—measures the degree of dispersion of data relative to the average; Coefficient of Variation (CV)—the ratio of the standard deviation to the mean, which compares the degree of dispersion between different data sets.

**Table 3:** Horizontal shift factors at the reference stress of 16 MPa under different aging times at 60 °C and 90% RH.

σ/MPa	60 °C, 90%RH			
	7 d	14 d	21 d	28 d
14 MPa	−0.33	−0.56	−0.48	−0.33
16 MPa	0	0	0	0
18 MPa	0.63	0.42	0.66	0.45
20 MPa	1.35	1.03	1.28	0.90
Shift factor equation	y = −4.41 + 0.28x	y = −4.20 + 0.26x	y = −4.68 + 0.30x	y = −3.26 + 0.21x

**Table 4:** Shift factors of PVC aged under a 60 °C, 90% RH environment with a reference stress of 16 MPa for varying durations.

Number of Days	7 d	14 d	21 d	28 d
Shift factor	0	2.12	5.68	7.40

## Data Availability

Data are contained within the article.
